# Editorial: Single-cell sequencing of the immune microenvironment in cancer

**DOI:** 10.3389/fimmu.2025.1660831

**Published:** 2025-07-15

**Authors:** David G. Coffey

**Affiliations:** Division of Myeloma, Sylvester Comprehensive Cancer Center, University of Miami, Miami, FL, United States

**Keywords:** single cell RNA (scRNA), hepatocellar carcinoma (HCC), endometrial cancer, spatial transcriptomics, T cell exhaustion

Cancer progression and treatment response are deeply influenced by the complex interplay between malignant cells and the immune microenvironment. Single-cell RNA sequencing (scRNA-seq) and spatial transcriptomics have emerged as transformative technologies for dissecting this complexity, offering unprecedented resolution into cellular heterogeneity and functional diversity within tumors. This Research Topic features three original research papers examining the tumor microenvironment (TME) in hepatocellular carcinoma (HCC) and one comprehensive review addressing scRNA-seq studies in endometrial cancer, offering relevant insights that extend across the broader field of cancer immunology.

HCC, the most prevalent form of primary liver cancer and a major cause of cancer-related mortality worldwide, has seen significant advances with the introduction of immune checkpoint inhibitor-based therapies (1). However, their benefit is limited to a subset of patients. A key determinant of therapeutic response is the TME, which profoundly shapes immune function and tumor evolution. Within the TME, CD8^+^ T cells often enter a state of exhaustion due to chronic antigen exposure and immunosuppressive signaling. This dysfunctional state, characterized by impaired effector activity and sustained expression of inhibitory receptors, presents a significant barrier to achieving durable immunotherapy responses (2). Understanding and reversing T cell exhaustion is therefore critical to improving immunotherapeutic outcomes in HCC. The three studies in this Research Topic illuminate novel mechanisms of immune exhaustion in HCC, leveraging single-cell and bulk RNA sequencing alongside spatial transcriptomics.

Jin et al. identified secreted phosphoprotein 1 (*SPP1)* expressing macrophages as key mediators of immune suppression. By using scRNA-seq data from HCC samples to deconvolute bulk RNA-seq profiles from a larger cohort, they quantified immune cell type abundance and demonstrated that *SPP1* ^+^ macrophages are associated with poor prognosis due to their ability to suppress CD8^+^ T cell proliferation. Importantly, they showed that inhibition of *SPP1* can reprogram macrophages toward a less suppressive phenotype, suggesting a viable therapeutic strategy for modulating macrophage function in HCC.

Expanding on the immunosuppressive mechanisms in HCC, Chen et al. explored the role of high-mobility group box 2 (*HMGB2)*, a member of the *HMGB* protein family involved in transcriptional regulation, DNA repair, and chromatin remodeling. Elevated *HMGB2* expression has been previously linked to a poor prognosis in HCC. Through a multi-omics approach, integrating scRNA-seq, bulk RNA-seq, spatial transcriptomics, and multiplex immunohistochemistry, they demonstrated that *HMGB2* fosters immune evasion by promoting T cell exhaustion. Their findings highlight *HMGB2*’s potential as both a prognostic marker and a therapeutic target in HCC.

Huang et al. turned their focus to microvascular invasion (MVI), an established risk factor for recurrence and poor outcomes in HCC. MVI is defined by the infiltration of malignant cells into the microvasculature surrounding the tumor. By integrating scRNA-seq and spatial transcriptomics, they identified a malignant cell subtype enriched for *MYC* signaling and strongly associated with MVI and adverse prognosis. These cells exhibited prominent crosstalk via macrophage migration inhibitory factor signaling. Using these features, the authors developed a machine-learning-based prognostic model, illustrating the clinical potential of combining high-resolution transcriptomic data with computational analytics.

The final contribution, a review by González-Martínez et al., surveyed the current landscape of scRNA-seq studies in endometrial cancer. They emphasized the cellular complexity of the TME across histological subtypes, drawing attention to the roles of tumor-associated macrophages, cancer-associated fibroblasts, and tumor-infiltrating lymphocytes. The authors also underscored several persistent challenges in the application of scRNA-seq, many of which extend to the broader field of tumor immunology. These include inconsistencies in tissue dissociation protocols, sequencing platforms, and parameter settings across studies. Computational variability further exacerbates the issue, with divergent approaches to quality control, data normalization, and cell annotation methods, particularly those used to distinguish between malignant and non-malignant cells. In addition, the limited number of cases analyzed, particularly among rare or aggressive histological subtypes, constrains the generalizability of findings. The authors advocate for standardization of both experimental and computational pipelines to improve reproducibility across studies and emphasize the need for larger, more diverse cohorts to capture the full spectrum of tumor heterogeneity.

Together, these studies demonstrate the unique power of single-cell and spatial transcriptomic technologies to elucidate the nuanced cellular and molecular dynamics of the immune microenvironment in cancer. By uncovering new mechanisms, identifying actionable targets, and informing clinical risk models, this work highlights how these cutting-edge approaches are poised to reshape cancer biology and drive more personalized and effective oncology care.
